# Supervised fine-tuning enhances unsupervised learning from 45 million amino acids in TCR and peptide sequences

**DOI:** 10.1093/bioinformatics/btag200

**Published:** 2026-04-24

**Authors:** Kewei Zhou, Kai Xu, Shaolong Lin, Silong Zhai, Huanxiang Liu, Xiaojun Yao

**Affiliations:** Centre for Artificial Intelligence Driven Drug Discovery, Faculty of Applied Sciences, Macao Polytechnic University, Rua de Luís Gonzaga Gomes, Macao, 999078, China; Centre for Artificial Intelligence Driven Drug Discovery, Faculty of Applied Sciences, Macao Polytechnic University, Rua de Luís Gonzaga Gomes, Macao, 999078, China; Centre for Artificial Intelligence Driven Drug Discovery, Faculty of Applied Sciences, Macao Polytechnic University, Rua de Luís Gonzaga Gomes, Macao, 999078, China; Centre for Artificial Intelligence Driven Drug Discovery, Faculty of Applied Sciences, Macao Polytechnic University, Rua de Luís Gonzaga Gomes, Macao, 999078, China; Centre for Artificial Intelligence Driven Drug Discovery, Faculty of Applied Sciences, Macao Polytechnic University, Rua de Luís Gonzaga Gomes, Macao, 999078, China; Centre for Artificial Intelligence Driven Drug Discovery, Faculty of Applied Sciences, Macao Polytechnic University, Rua de Luís Gonzaga Gomes, Macao, 999078, China

## Abstract

**Motivation:**

T cell receptor (TCR) and peptide interactions (TPI) are one of the most important parts of T cell immunity. Experimental identification of TPI is time-consuming and labor-intensive; therefore, it is necessary to develop computational prediction method that exploit existing data to predict TPI.

**Results:**

We use huge TCR and peptide sequences to pre-train two language models (∼152M parameters), respectively, and integrate them into a sequence-based only prediction framework (i.e. RoBERTcr) with supervised fine-tuning (SFT). Visualization of amino acids embedding from pre-trained language model (PLM) shows biochemical clusters based on different properties, and our PLMs outperform existing protein language models (i.e. ESM and ProtTrans) under the same condition. RoBERTcr achieved higher performance than other state-of-the-art methods based on structures or sequences without dataset bias. The visualization of attention from our framework implies valuable spatial information that residues in TCR contacting peptides are the key to their interaction.

**Availability:**

RoBERTcr is free available at https://fca_icdb.mpu.edu.mo/robertcr/ and https://doi.org/10.5281/zenodo.18043054.

## Graphical Abstract

**Graphical Abstract:**
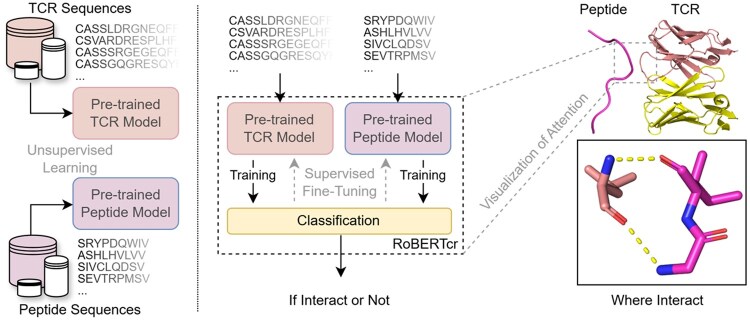

## 1 Introduction

T cells compose the key to adaptive immune response and the immunity they mediated, which protects infection and cancer, is integral to human health (Mhanna et al. 2024, Raynor and Chi 2024). T cell immunity is a three-signal model incorporating antigen presentation, co-stimulation, and cytokines (Giles et al. 2023). In the procedure of antigen presentation, a major histocompatibility complex (MHC) presents antigen peptide processed in antigen-presenting cell to its surface for T cell receptor (TCR) combining (Pishesha et al. 2022). Despite a few cases of cross-recognize, the combination of MHC, antigen peptide and TCR is such highly specific that TCR immunotherapy has much potential in precision medicine (Gao et al. 2022, Keeton et al. 2022, Tarke et al. 2022, Baulu et al. 2023). The role of the TCR in developing new therapies has driven researchers to decode which T cells recognize antigen peptide. However, the cost and experimental limitations restrict the possibility of exploring the boundaries of all binding pairs. According to Soto et al. the size of individual human recombined and expressed TCR is between 5 and 21 million clonotypes and 3 individuals share 8% of TCRβ or 11% of TCRα chain clonotypes (Soto et al. 2020). Therefore, the development of time and money-saving methods to predict TPI *in silico* will contribute to a deep understanding of the TCR in surveillance and response to disease.

One of the key steps of computational methods is conducting feature engineering or input embedding, which converts sequences into vectors. Traditional machine learning methods usually use one-hot encoding or BLOSUM matrix integrating with random forest or convolutional neural network (Springer et al.2020, Montemurro et al. 2021, Moris et al. 2021, Xu et al. 2021). However, this situation was overshadowed after the protein language model (PLM) came up. The most frequently used PLM recently is ESM2 which was trained with up to 15 billion parameters on experimental and high-quality predicted structures and found information about atomic-level structure (Lin et al. 2023). ESM2 serial models have shown their powerful capacity for sequence embedding in many downstream tasks (Pham et al. 2023, Ito et al. 2025, Kshirsagar et al. 2025, Sun et al. 2025, Mou et al. 2026).

It is noteworthy that those PLMs were pre-trained on natural protein sequences ranging up to thousands of amino acids while the length of TCR sequences (restricted to CDR3α or CDR3β) is usually less than 25 and so are peptides (Goncharov et al. 2022). The number of proteins collected in UniProtKB is 253 635 358 (release 2025_03) and PLMs are usually trained on redundancy reduced clusters such as UniRef50 (Rives et al. 2021, Elnaggar et al. 2022, Lin et al. 2023, UniProt et al. 2024). As we focus on TCR sequencing data, it contains approximately 290 314 598 sequences (duplicate reduced from 691 744 135 as of TCRdb2.0) which is more than whole proteins with higher sequence identity (Montemurro et al. 2021). Since the sequences of TCR and peptide are different from what existing PLMs trained on, we pre-train two PLMs focusing on TCR and peptide, respectively, and integrated them (i.e. RoBERTcr) to predict TPI. Through a series of experiments, we observed that our sequence-based only model outperforms existing structure-based methods whatever the dataset is. Under the same conditions, embedding extracted from our model is better than existing PLMs trained on natural protein sequences. Interestingly, model visualization shows that our model learns and focuses on amino acids that are spatial contacting in interaction. Furthermore, we build a user-friendly web server for one-stop online prediction, key residue visualization, and high-throughput mutation screen taking advantage of RoBERTcr (processing nearly 5k pairs per second).

## 2 Methods

### 2.1 Pre-training data and model

TCR sequencing data is extracted from TCRdb2.0. It contains 691 744 135 sequences (https://guolab.wchscu.cn/TCRdb2) (Yue et al. 2025). We download all original sequences and after dropping duplicates, it remains 290 314 598 sequences ranging between 11 and 24 in length. Then we use cd-hit (v4.8.1 from https://github.com/weizhongli/cdhit) to reduce redundancy (Fu et al. 2012). Different identity cut-offs are tested, and we choose 75% (1 782 927 remains) as the final value. Peptide sequences are extracted from TransPHLA dataset, and the number is 1 366 389 (https://github.com/a96123155/TransPHLA-AOMP) (Chu et al. 2022).

We use a masked language model called RoBERTa which has the same architecture as BERT but uses a different pretraining scheme including dynamic masking, sentence packing, larger batches and byte-level byte pair encoding vocabulary (Zhuang et al. 2021). We start with original model configuration (https://huggingface.co/FacebookAI/roberta-base) and do some hyper parameter searching work to find out the best for TCR sequencing data. The final trainable parameters are 152 308 768 and we initialize it from the Hugging Face training library (v4.51.1). This experiment is conducted on a HPC node with dual Intel Xeon Platinum 8468 CPUs, 2 TB system memory and an NVIDIA H100 80G HBM3 GPU. The pre-training time of 1 782 927 TCR and 1 366 389 peptide sequences take about 9 hours and 6 hours under 64 epochs, respectively. All pre-training details and model configs can be found at https://huggingface.co/keiwoo. It is also mentioned that we use BF16 to speed up approximately 4 times than the commonly used FP32 precision.

### 2.2 Supervised fine-tuning data and model

We use the training and testing data from methods that have been peer reviewed and published to implement fair comparisons. If the data is explicitly accessible from the repository, we will use it directly (DLpTCR, TARB-BERT and DAISY). Some methods (TRAP, TEIM and TEPCAM), however, process training and testing data during their programs running; we have no choice but to add some code into their open-source programs to export the training and testing data. Those procedures are handled carefully to make sure the datasets are the same as they should. All methods are reproduced, retrained and evaluated on these matched datasets according to their scripts.

After getting training and testing data, we stack two pre-trained models and design a classification head to integrate embeddings and predict binding possibilities. The classification head accepts embeddings from two pre-trained models [dimension of (CLS + *N* + SEP) * 1024, where *N* is the length of sequence and CLS and SEP are automatically added by the model and dropped later]. The embeddings are averaged to dimensions of 1024 and concatenated with their Hadamard product to generate a vector of 3072 dimensions. After a fully connected layer, the classification head will return binding possibilities between 0 and 1 (a possibility ≥ 0.5 was thought to be interaction) and compute loss to fine-tune the pretrained parameter. This experiment is conducted on a HPC node with dual Intel Xeon Platinum 8468 CPUs, 2 TB system memory and an NVIDIA H100 80G HBM3 GPU. The supervised fine-tuning takes about 15 minutes on 214 779 TCR-peptide pairs (randomly split train set) or 18 minutes on 232 135 TCR-peptide pairs (zero-shot train set).

### 2.3 Performance evaluations

To provide a comprehensive performance assessment for models, the precision-recall (PR) curve is plotted, and the area under the PR curve (AUPR) is also calculated to quantify the performance. In the meantime, the receiver operating characteristic (ROC) curve, which plots the true positives rate value against the false positives rate value at different thresholds, and the corresponding area under ROC curve (AUROC) are also employed for performance assessment. Accuracy, Precision, Recall, and F1 are calculated by scikit-learn Python package directly.

### 2.4 Amino acid visualization

T-SNE is used to reduce and visualize high dimensions of each amino acid extract from a pre-trained model without SFT. Firstly, the last hidden state of 70 827 positive TCR sequences from VDJdb are extracted and their dimensions are (CLS + *N* + SEP) * 1024 (where *N* is the length of sequence), which means that the dimension of each amino acid is 1*1024 in other words. Then, we add and mean all 1*1024 matrixes according to 20 different amino acids. Finally, those 1*1024 matrixes are reduced by t-SNE and draw a scatter plot.

### 2.5 Ablation study

We design parameter-random models (which means no pre-training) and parameter-froze models (which means no SFT) to test pre-training and fine-tuning impact but not including the classification head. The parameter-random models initiate their parameters from random state before SFT. The parameter-froze models load parameters from pre-training but will not update them during SFT. To evaluate alternative interaction modules, we additionally test bilinear pooling and cross-attention fusion. In bilinear pooling, TCR and peptide embeddings are combined through a learnable bilinear transformation to model pairwise feature interactions with higher expressiveness than element-wise fusion. In cross-attention, TCR sequence embedding is used as query and the peptide as key and value (in both directions), and the attended representations are pooled and concatenated for classification. We also extract ESM (esm1b_t33_650M_UR50S, esm2_t33_650M_UR50D) embedding and averages them to dimensions of 1280 as well as ProtT5 (ProtT5-XL-UniRef50) to dimensions of 1024 according to their instrument. Those embeddings are input into the same classification head as mentioned above (we adjust the number of input features from fully connected layer to fit ESM embedding). The evaluations are calculated to assess the performance drop.


$$\frac{\mathrm{Ablation}\;\mathrm{Performance}-\mathrm{Origin}\;\mathrm{Performance}}{\mathrm{Origin}\;\mathrm{Performance}}\times 100\%$$

where ‘Origin Performance’ represents our entire model performance and ‘Ablation Performance’ can also represent the performance of ESM and ProtT5.

### 2.6 Interpretability study

The attentions are firstly extracted from last layer of PLM and mean to length of sequence (except CLS and SEP) to plot heatmap. The structure of MEL5-HLA-A*02:01-AAGIGILTV complex (PDB ID: 6EQA), HCV1406-HLA-A*02:01-KLVALGINAV complex (PDB ID: 5JZI), TCR589-HLA-B*35:01-IPLTEEAEL complex (PDB ID: 6BJ2) and the clone 12 TCR-HLA-B*35:42-IPSINVHHY complex (PDB ID: 4QRR) are downloaded from RCSB protein data bank and visualized by PyMOL software with reference to original research articles (Pellicci et al. 2014, Wang et al. 2017, Sibener et al. 2018, Madura et al. 2019).

## 3 Results

### 3.1 Pre-trained model encodes biochemical properties

**Figure 1:**
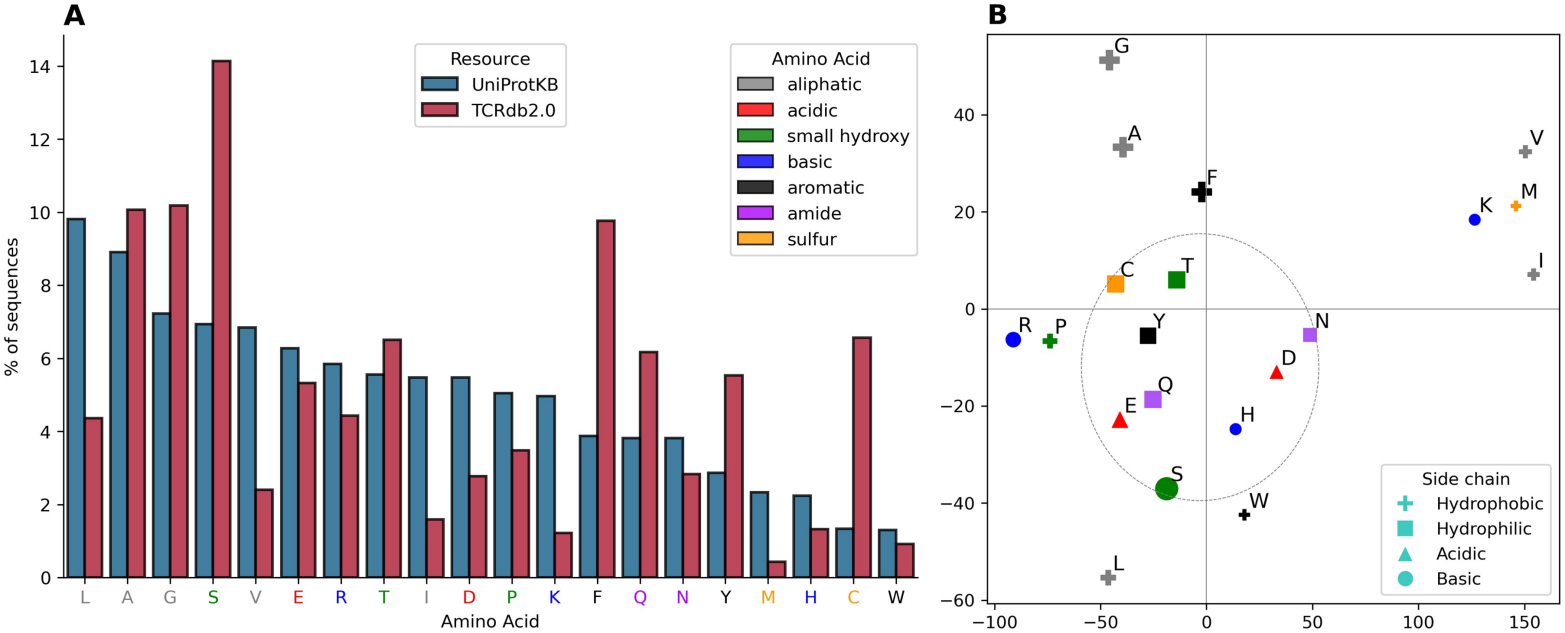
Statistics and t-SNE dimension reduction of 20 amino acids. (A) Plots the frequences of 20 amino acids from UniProtKB (v2025_03) and TCRdb2.0. The statistics of UniProtKB were obtained from UniProtKB statistics website directly and TCRdb was calculated from 290 314 598 sequences. (B) Plots the dimension reduction of 20 amino acids representation from the last hidden output of a pre-trained model. The frequency of amino acids is manifested as different legend sizes.

UniProt is the world’s leading high-quality and comprehensive resource of protein sequence and functional information, and it has amino acid composition statistics (Table S1) from 253 635 358 (release 2025_03) sequences in UniProtKB. We statistic 290 314 598 TCR sequencing data as the order of UniProt which is so different from it as Fig. 1A shows, despite the IMGT defined that CDR3 starts from C and ends at F which may cause high frequencies themselves. The frequences of M (−82%), K (−76%), I (−71%) and V (−64%) drop sharply while S (+104%), Y (+93%) and Q (+61%) increase. This situation may cause different pre-training results. To investigate if our PLM of TCR learned amino acid properties in its representations, we project the final embedding layer of the model into two dimensions with t-distributed stochastic neighbor embedding (t-SNE). In Fig. 1B, the structure of the embedding space reflects biochemical interchangeability with distinct clustering of hydrophobic (outer circle) and polar residues (inner circle). M, K, I and V are also far away from others which indicate that they may have side effect in TCR considering their frequency and long and straight-chain with steric effects. Inspired by these results, we get PLMs of TCR and peptide and use them to predict interaction as follows.

### 3.2 Integration of supervised fine-tuning models outperforms existing methods

**Figure 2:**
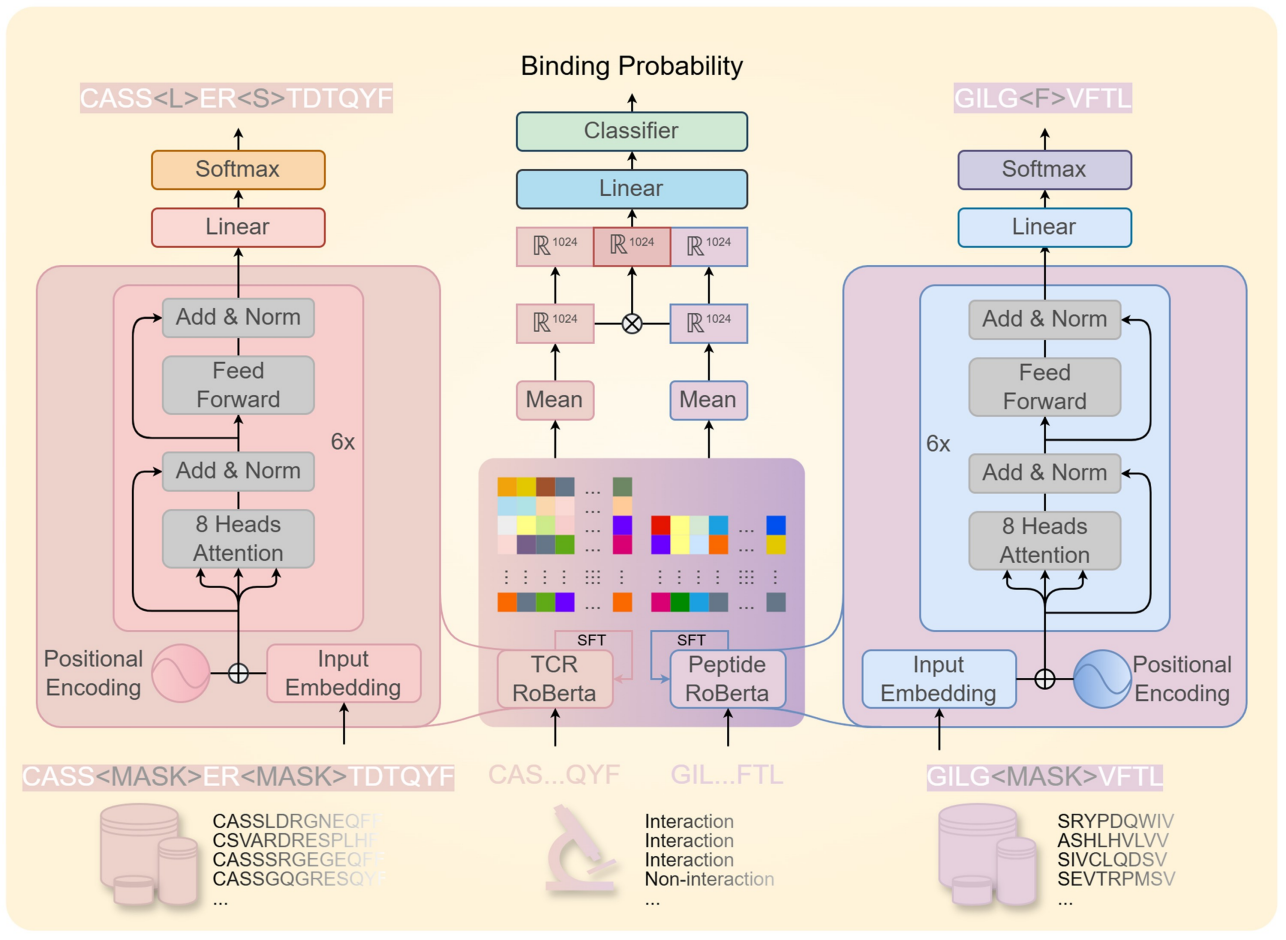
The schematic diagram of RoBERTcr. Firstly, TCR and peptide sequences from databases were extracted for pre-trained language models (left and right part). Then, these two pre-trained models were stacked together to construct a supervised fine-tuning model which outputs the embeddings of TCR and peptide (middle part). Finally, the embeddings were fused to generate prediction score between 0 and 1 (a prediction score ≥ 0.5 corresponded to a positive interaction).

We design a computational framework (Fig. 2) based on our PLMs for the prediction of TPI based on sequence only. This framework has two cores, TCR and peptide PLMs which are masked language models called RoBERTa that pre-trained under the strategy of predicting the randomly masked amino acids (left and right part of Fig. 2) (Zhuang et al. 2021). After pre-training, they accept TCR and peptide sequences and embed them, respectively. Then, we extract the final hidden layer outputs and mean them to 1024 dimensions of embedding. After that, those two embeddings and their Hadamard product are concatenated to a vector of 3072 dimensions considering a separated and fused feature. Finally, the vector of 3072 is inputted to a full connection layer before classification. When training, the two pre-trained models are supervised fine-tuning (SFT) for their parameters simultaneously.

**Figure 3:**
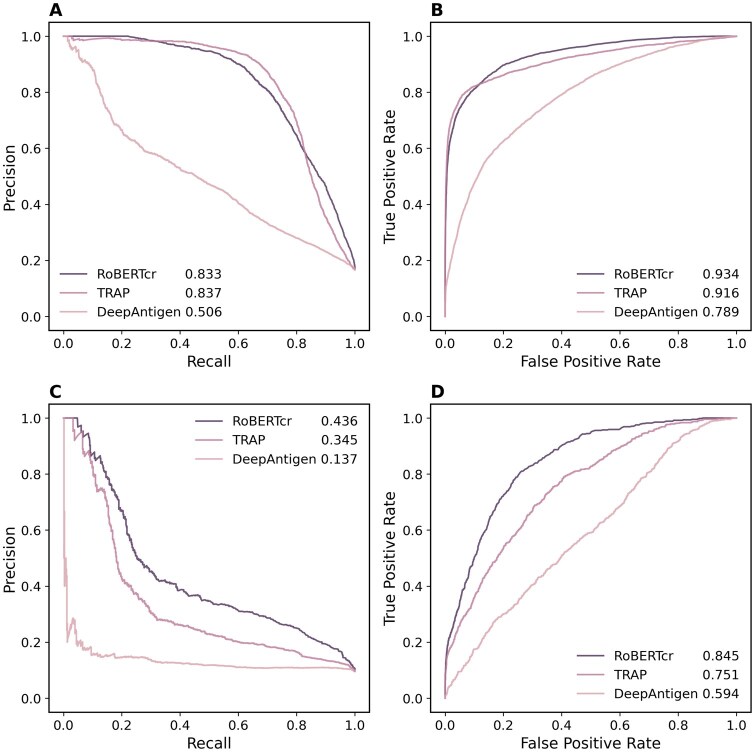
Performance comparison with two recent methods. (A) Plots the PR curves on the randomly split test set (positive and negative sample ratio is 1:5), while (B) plots the ROC curves. (C) Plots the PR curves on the zero-shot test set (positive and negative sample ratio is 1:0), while (D) plots the ROC curves. Parameters in the legends denote the corresponding area under curve values.

We compare our method with the most recent state-of-the-art TRAP, a contrastive learning-enhanced framework by aligning structural and sequence features as well as DeepAntigen, a graph convolutional network-based framework identifying at the atomic level (Ge et al. 2025, Que et al. 2025). The results are shown in Fig. 3 and Table S2. As can be seen, our method outperforms DeepAntigen on both test sets. The performances of TRAP are slightly higher (+0.004, *P *< .05) than ours on PR but lower (−0.018, *P *< .05) on ROC (Fig. 3A and B, randomly split test set, the ratio of positive and negative sample is 1:5) meaning that TRAP is likely to predict interaction rather than non-interaction which will cause higher false positives but get high performance on relative easy dataset indeed as their claim. When it comes to the difficult dataset (Fig. 3C and D, zero-shot test set, the ratio of positive and negative samples is 1:10), however, the performances of TRAP dropped more than RoBERTcr (−0.091 on PR and −0.094 on ROC; both *P *< .01). We also compare RoBERTcr with several methods with self-constructed datasets to test its generalization and nearly outperformed on all datasets at the same evaluation (Table S3).

### 3.3 Ablation and interpretability study

**Figure 4:**
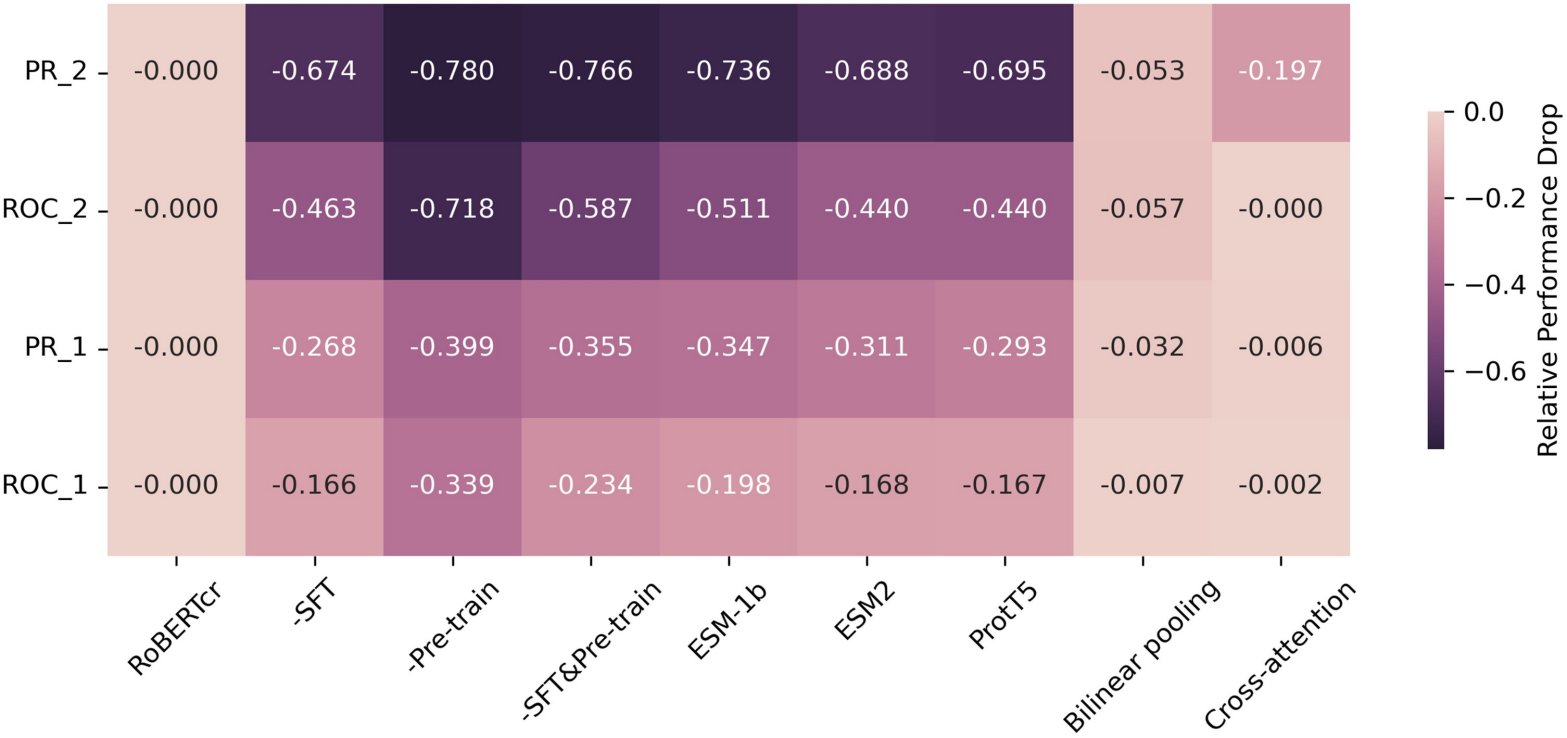
Ablation study of RoBERTcr. PR and ROC correspond to the area under them. Rand corresponds to the randomly split test set while Zero corresponds to the zero-shot test set from TRAP. RoBERTcr corresponds to its original performance. – corresponds to a model without that procedure. ESM-1b, ESM2 and ProtT5 correspond to performances by replacing RoBERTcr embedding with theirs under the same prediction framework. Bilinear pooling and cross-attention correspond to performance replacing Hadamard product with them. The formula for calculating relative performance drops is available in the Methods section.

To assess the contribution of different parts of our method, we design three tasks by random initiation (no pre-training), parameter frozen (no SFT), and both (no pre-training and SFT). What’s more, we implement the same computational framework on famous PLMs by replacing model embedding with them (esm1b_t33_650M_UR50S, esm2_t33_650M_UR50D and ProtT5-XL-UniRef50). The results show (Fig. 4) that pre-training and SFT have different importance to our computational framework whose performance is dropping sharply without pre-training. Especially, this drop is more significant on difficult test sets (maximum 78.0% of PR on zero-shot test set versus 39.9% on randomly split). It is also found that the performance of our PLMs without SFT is even slightly better than existing PLMs when using the same prediction framework. This result is consistent with our analysis in Fig. 1 that our models have different pre-train dataset distributions from them which generate better animo acid embedding of TCR and peptide sequences. That is said that our PLMs are more suitable for TPI tasks than those pre-trained on natural protein sequences. Also, the different embedding integration techniques are compared including bilinear pooling and cross-attention. While these techniques take effect in some cases, they do not improve AUPR/AUROC relative to the Hadamard product but increase computational overhead (more training parameters and slower inference) in our benchmarks (Chen et al. 2025, Cui et al. 2025, Gui et al. 2026, Yan et al. 2026).

**Figure 5:**
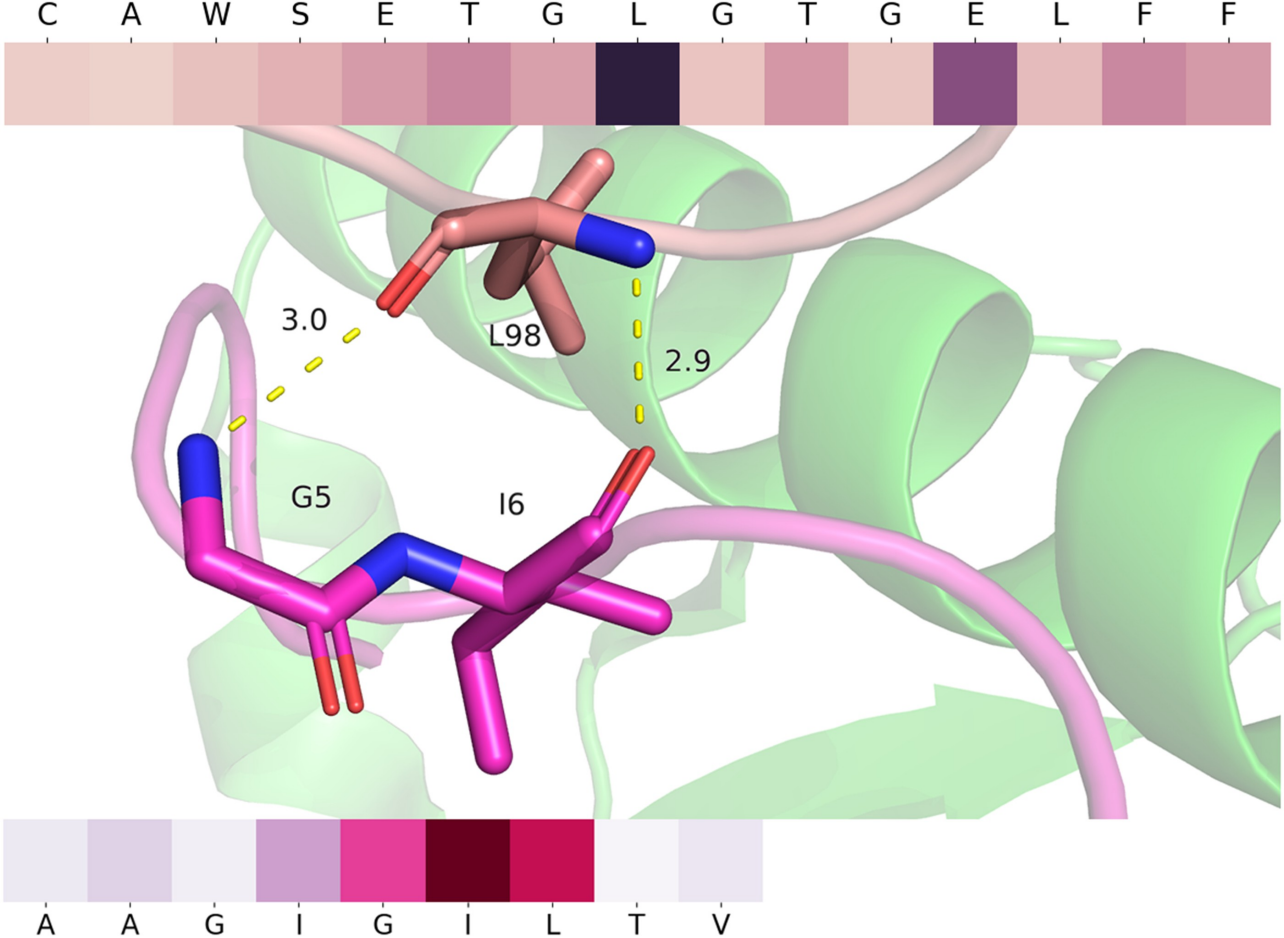
Crystallographic structure of MEL5-HLA-A*02:01-AAGIGILTV complex (PDB ID: 6EQA). The colors of MEL5-CDR3β, HLA-A*02:01 and AAGIGILTV are light pink, green, and magenta. The hydrogen bonds are shown as yellow dash, and the unit of measurement is Å. The heatmap is the visualization of attention where the deeper color means more attention on it, and the tick is the sequence of CDR3β and peptide.

A few residues play a key role during the interaction of TCR and peptide and the identification of these residues has the potential of affinity optimization and sequence design (Shah et al. 2021). Our PLMs based on attention could evaluate contributions from different amino acids and provide an interpretable signal of which positions may be influential. We take a melanoma surface antigen for example (three more cases available at Figs. S1–S3), AAGIGILTV is a nonapeptide at the melanoma cell surface represented by HLA-A*02:01 and interacting with MEL5 TCR. The structure of the MEL5-HLA-A*02:01-AAGIGILTV complex (PDB ID: 6EQA) revealed an induced fit mechanism of antigen recognition (Madura et al. 2019). After predicting (score 0.999) and extracting weight inside model attention, we observed that RoBERTcr assigns relatively high attention to Leu-98β (CAWSETGLGTGELFF) and Ile-6 (AAGIGILTV) which is consistent to the result of crystallographic structure (Fig. 5) that Leu-98β makes hydrogen bonds with Gly-5 and Ile-6 (“GIGI” motif in the center of the AAGIGILTV). If we mutate L with the other 19 amino acids, only three mutations will interact in our prediction indicating that mutations significantly affect prediction scores and the mutation to M, T and F may have the potential to become novel interactive TCR sequences.

### 3.4 Web server

As far as we know, only DLpTCR provides web server in the past 5 years of publications (http://jianglab.org.cn/DLpTCR/Server) (Xu et al. 2021). DLpTCR only accepts format-fixed file uploading and email address is required to receive result. To promote our method and facilitate researchers who are not familiar with coding, we design an efficient, user-friendly, and privacy-protected website with modern design to provide fast and convenient prediction of TPI (https://fca_icdb.mpu.edu.mo/robertcr/). Users can directly paste their sequences to the input boxes, and we will check format automatically before submitting to server and give error information below the input boxes if there is anything wrong with their sequences. The server will accept the same number of TCR and peptide to predict one-on-one which is alignment mode. We also provide a combination mode which does not restrict the same number of inputs if the user is likely to do cross-prediction (screen every TCR on every peptide, etc.). After prediction, the website will show downloadable results including sequences and bind possibilities. It also provides visualization of key residues involved in interaction. Benefit from our high efficiency model (processing nearly 5k pairs per second), we also allow users to screen 4 mutations at most on their interesting sequences which will predict 194 481 (21^4^, including insertion and deletion mutation) TCRs on specific peptide.

### 3.5 Limitations

Although RoBERTcr shows strong efficiency and competitive accuracy, several limitations should be noted. First, our framework is sequence-based and does not explicitly model structural context; therefore, performance may decrease in cases where binding is highly dependent on dynamic conformation. Second, while we evaluate on random-split and zero-shot settings, the available benchmark datasets are still limited compared with the real diversity of the immune repertoire, which may affect generalization to rare or out-of-distribution TCR-peptide pairs. Finally, predictions from RoBERTcr, including mutation screening results, are hypothesis-generating and still require experimental validation.

## Supplementary Material

btag200_Supplementary_Data
