# Supplementary material for: Supervised fine-tuning enhances unsupervised learning from 45 million amino acids in TCR and peptide sequences
Source: Bioinformatics. 2026 Apr 24;42(5):btag200. doi: 10.1093/bioinformatics/btag200 (PMC13175252; doi:10.1093/bioinformatics/btag200)
Supplement: btag200_Supplementary_Data [file btag200_supplementary_data.zip › 01-May-2026_020824_supplementary_material.docx]

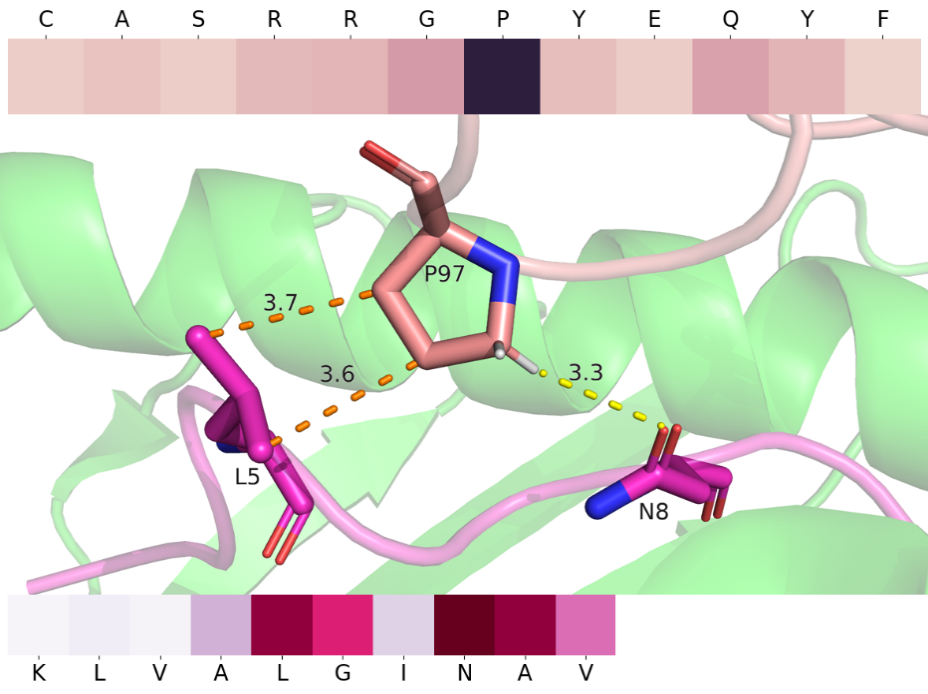


Fig. S1: Crystallographic structure of HCV1406-HLA-A*02:01-KLVALGINAV complex (PDB ID: 5JZI). The colors of HCV1406-CDR3β, HLA-A*02:01 and KLVALGINAV are light pink, green, and magenta. Hydrogen bond and hydrophobic interactions are shown as yellow and orange dash, respectively. The visualization of attention is shown as heatmap. Nonstructural protein 3 peptide:1406-1415 (KLVALGINAV) is from the hepatitis C virus (HCV) presented by HLA-A*02:01 and interacting with HCV1406 TCR (Wang 2017). The prediction score from RoBERTcr is 0.982 and the visualization of attention is consistent to the result of crystallographic structure that Leu-5 (KLVALGINAV) fits into a large hydrophobic cleft formed by residues in CDR1α, CDR3α, and CDR3β. Asn-8 (KLVALGINAV) also forms hydrogen bond with Pro-97β (CASRRGPYEQYF).


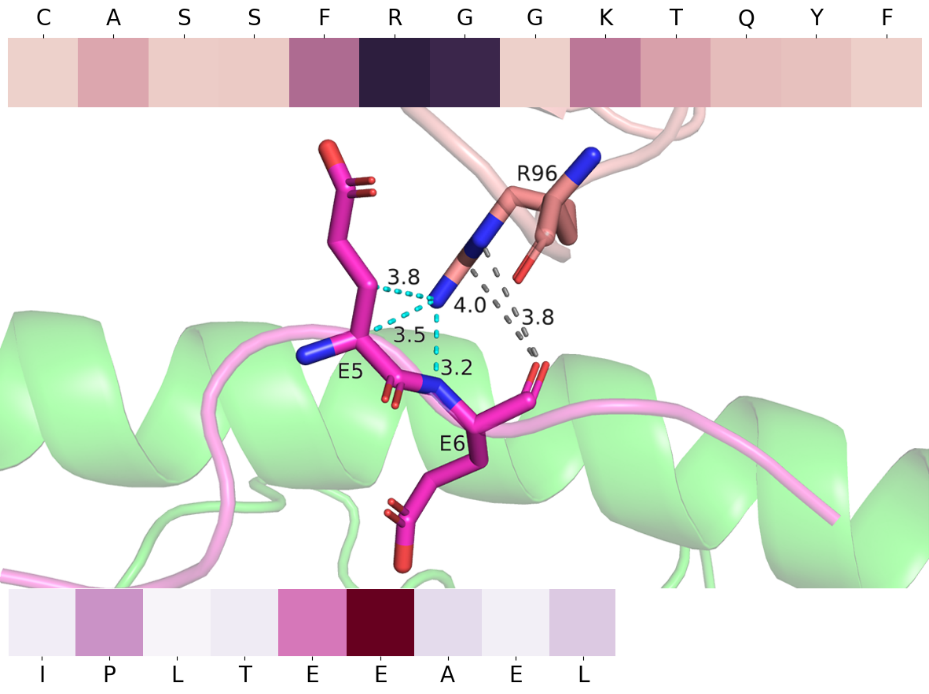


Fig. S2: Crystallographic structure of TCR589-HLA-B*35:01-IPLTEEAEL complex (PDB ID: 6BJ2). The colors of TCR589-CDR3β, HLA-B*35:01 and IPLTEEAEL are light pink, green, and magenta. Van der Waals interactions and salt bridges are shown as gray and cyan dash, respectively. The visualization of attention is shown as heatmap. Pol B35 peptide:448-456 (IPLTEEAEL) is from the human immunodeficiency virus (HIV) presented by HLA-B*35:01 and interacting with TCR589 (Sibener 2018). The prediction score from RoBERTcr is 0.993 and the visualization of attention is consistent to the result of crystallographic structure that Arg-96β (CASSFRGGKTQYF) forms Van der Waals interactions and salt bridges to Glu-5 and 6 (IPLTEEAEL) which is most of the binding interface from CDR3β.


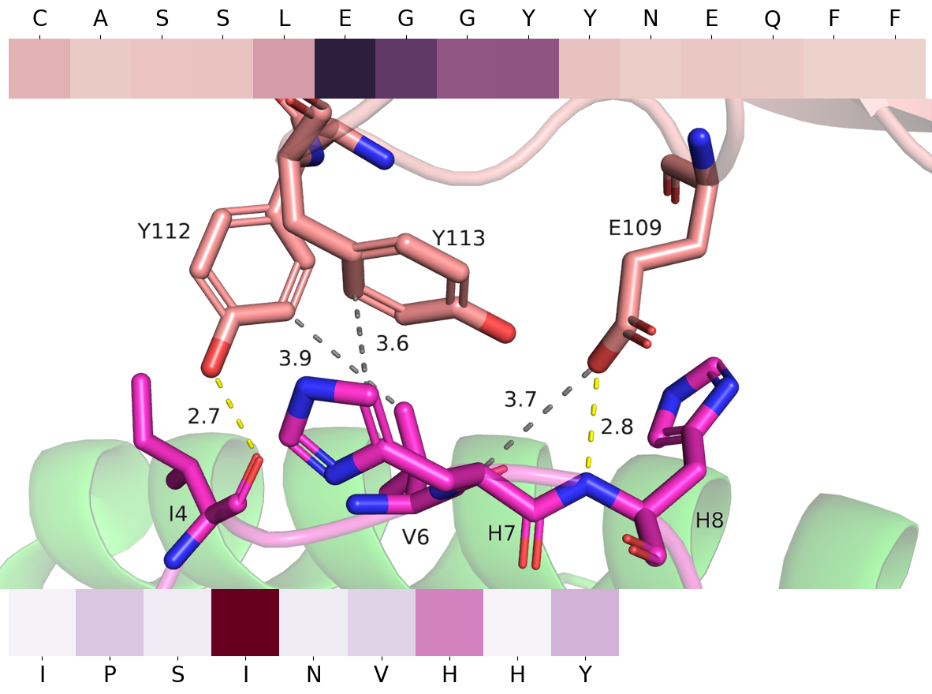


Fig. S3: Crystallographic structure of the clone 12 TCR-HLA-B*35:42-IPSINVHHY complex (PDB ID: 4QRR). The colors of the clone 12 TCR-CDR3β, HLA-B*35:42 and IPSINVHHY are light pink, green, and magenta. Van der Waals interactions and hydrogen bond are shown as gray and yellow dash, respectively. The visualization of attention is shown as heatmap. IPSINVHHY (IPS) is from the cytomegalovirus (CMV) presented by HLA-B*35:42 and interacting with the clone 12 TCR (Pellicci 2014). The prediction score from RoBERTcr is 0.989 and the visualization of attention is consistent to the result of crystallographic structure that the CDR3β loop forms most of the interactions with the IPS contacting the Ile-4, Val-6, His-7 and His-8 (IPSINVHHY). Tyr-112β (CASSLEGGYYNEQFF) inserts its aromatic ring between the main chain of the Ile-4 and Val-6, whereas Tyr-113β (CASSLEGGYYNEQFF) sits atop the His-7 and contacts theVal-6. Furthermore, Glu109β contacted His-7 and hydrogen bonded to the main chain of His-8.

Table S1: Composition in percentage for the UniProtKB Release 2025_03^a^ and TCRdb2.0

| Total of 253,635,358^b^/290,314,598 entries | | | |
| --- | --- | --- | --- |
| Ala (A) 8.91/10.06 | Gln (Q) 3.81/6.17 | Leu (L) 9.80/4.36 | Ser (S) 6.94/14.14 |
| Arg (R) 5.85/4.43 | Glu (E) 6.26/5.33 | Lys (K) 4.97/1.22 | Thr (T) 5.56/6.51 |
| Asn (N) 3.81/2.83 | Gly (G) 7.22/10.18 | Met (M) 2.32/0.43 | Trp (W) 1.30/0.91 |
| Asp (D) 5.47/2.78 | His (H) 2.24/1.32 | Phe (F) 3.87/9.76 | Tyr (Y) 2.87/5.53 |
| Cys (C) 1.33/6.56 | Ile (I) 5.48/1.59 | Pro (P) 5.04/3.48 | Val (V) 6.83/6.83 |

The percentage of UniProtKB and TCRdb2.0 are splitted by slash.

a: https://ftp.ebi.ac.uk/pub/databases/uniprot/previous_releases/release-2025_03/knowledgebase/UniProtKB_TrEMBL-relstat.html

b: https://ftp.ebi.ac.uk/pub/databases/uniprot/previous_releases/release-2025_03/relnotes.txt

Table S2: Performance comparison with two recent methods

| Method | Area under PR curve | | Area under ROC curve | |
| --- | --- | --- | --- | --- |
|  | Randomly | Zero-shot | Randomly | Zero-shot |
| RoBERETcr | 0.833±0.002 | **0.436±0.008** | **0.934±0.002** | **0.845±0.009** |
| TRAP | **0.837±0.007**** | 0.345±0.041** | 0.916±0.011* | 0.751±0.044* |
| DeepAntigen | 0.506±0.016*** | 0.137±0.007*** | 0.789±0.006*** | 0.594±0.006*** |

The mean and standard deviation of 5-fold cross validation. *, ** and *** indicate that the statistical significances of paired t-test (two-sided) compared to corresponding result are *p* < 0.05, *p* < 0.01 and *p* < 0.001, respectively. Bold text indicates the best value for that column.

Table S3: Comparison of different methods on their own datasets.

| Method (dataset name) | AUROC | AUPR | Accuracy | F1 | Recall | Precision |
| --- | --- | --- | --- | --- | --- | --- |
| DLpTCR (Xu 2021) (pTCRβ-ensemble) | 0.90/0.94 | 0.90/0.94 | 0.81/0.86 | 0.83/0.86 | 0.91/0.86 | 0.76/0.86 |
| DLpTCR (Independent I) | 0.92/0.93 | 0.92/0.93 | 0.84/0.84 | 0.85/0.85 | 0.92/0.86 | 0.79/0.83 |
| TEIM (Peng 2023) | 0.71/0.75 | 0.43/0.48 | 0.85/0.85 | 0.30/0.39 | 0.19/0.29 | 0.69/0.60 |
| TEPCAM (Chen 2024) | 0.68/0.68 | 0.68/0.70 | 0.62/0.63 | 0.63/0.63 | 0.62/0.63 | 0.64/0.63 |
| TARB-BERT (Zhang 2023) (Te-S1) | 0.84/0.84 | 0.88/0.88 | 0.67/0.70 | 0.72/0.74 | 0.88/0.84 | 0.62/0.66 |
| TARB-BERT (Te-S2) | 0.90/0.95 | 0.89/0.96 | 0.65/0.74 | 0.74/0.79 | 0.97/0.98 | 0.59/0.66 |
| TARB-BERT (Te-S3) | 0.81/0.82 | 0.85/0.86 | 0.62/0.68 | 0.70/0.73 | 0.88/0.85 | 0.58/0.64 |
| TARB-BERT (Te-S4) | 0.93/0.94 | 0.94/0.96 | 0.73/0.74 | 0.78/0.79 | 0.95/0.94 | 0.66/0.68 |
| DAISY (Yuan 2025) (Seen-Pair) | 0.91/0.94 | 0.93/0.96 | 0.84/0.83 | 0.84/0.85 | 0.84/0.92 | 0.85/0.78 |
| DAISY (Unseen-TCR) | 0.90/0.93 | 0.92/0.95 | 0.84/0.81 | 0.84/0.83 | 0.83/0.91 | 0.84/0.76 |
| DAISY (Unseen-Epitope) | 0.91/0.93 | 0.93/0.95 | 0.84/0.82 | 0.84/0.84 | 0.84/0.91 | 0.85/0.78 |
| DAISY (Unseen-Pair) | 0.90/0.93 | 0.93/0.95 | 0.83/0.81 | 0.83/0.83 | 0.82/0.88 | 0.84/0.77 |

The score on the right of slash is ours.

References

Chen J, Zhao B, Lin S, *et al.* TEPCAM: prediction of T-cell receptor-epitope binding specificity via interpretable deep learning. *Protein Sci* 2024; **33**: e4841.

Peng X, Lei Y, Feng P, *et al.* Characterizing the interaction conformation between T-cell receptors and epitopes with deep learning. *Nat Mach Intell* 2023; **5**: 395–407.

Yuan Y, Chen J, Zhang Y, *et al.* A biologically informed vision-guided framework for interpretable T cell receptor-epitope binding prediction. *Adv Sci* 2025; **1**: e12544.

Zhang J, Ma W and Yao H. Accurate TCR-pMHC interaction prediction using a BERT-based transfer learning method. *Brief. Bioinform* 2023; **25**: bbad436.
